# Biokinetic and dosimetric aspects of ^64^CuCl_2_ in human prostate cancer: possible theranostic implications

**DOI:** 10.1186/s13550-018-0373-9

**Published:** 2018-03-01

**Authors:** Sergio Righi, Martina Ugolini, Gianluca Bottoni, Matteo Puntoni, Massimiliano Iacozzi, Francesco Paparo, Manlio Cabria, Luca Ceriani, Monica Gambaro, Luca Giovanella, Arnoldo Piccardo

**Affiliations:** 1 Medical Physics Department, E.O. Galliera Hospital, Genoa, Italy; 2 Department of Nuclear Medicine, Galliera Hospital, Mura delle Cappuccine 14, 16128 Genoa, Italy; 3 Clinical Trial Unit, Office of the Scientific Director, Galliera Hospital, Genoa, Italy; 4 Department of Radiology, E.O. Galliera Hospital, Genoa, Italy; 5 Department of Nuclear Medicine, Oncology Institute of Southern Switzerland, Bellinzona, Switzerland

**Keywords:** ^64^CuCl_2_, Prostate cancer, Elderly, Kinetics and dosimetry, Theranostic

## Abstract

**Background:**

The aim of the present study is to evaluate the kinetics and dosimetry of ^64^CuCl_2_ in human prostate cancer (PCa) lesions.

We prospectively evaluated 50 PCa patients with biochemical relapse after surgery or external beam radiation therapy. All patients underwent ^64^CuCl_2_-PET/CT to detect PCa recurrence/metastases. Volumes of interest were manually drawn for each ^64^CuCl_2_ avid PCa lesion with a diameter > 1 cm on mpMRI in each patient. Time-activity curves for all lesions were obtained. The effective and biological half-life and the standard uptake values (SUVs) were calculated. Tumour/background ratio (TBR) curves as a function of time were considered. Finally, the absorbed dose per lesion was estimated.

**Results:**

The mean effective half-life of ^64^CuCl_2_ calculated in the lymph nodes (10.2 ± 1.7 h) was significantly higher than in local relapses (8.8 ± 1.1 h) and similar to that seen in bone metastases (9.0 ± 0.4 h). The mean ^64^CuCl_2_ SUV_max_ calculated 1 h after tracer injection was significantly higher in the lymph nodes (6.8 ± 4.3) and bone metastases (6.8 ± 2.9) than in local relapses (4.7 ± 2.4). TBR mean curve of ^64^CuCl_2_ revealed that the calculated TBR_max_ value was 5.0, 7.0, and 6.2 in local relapse and lymph node and bone metastases, respectively, and it was achieved about 1 h after ^64^CuCl_2_ injection. The mean absorbed dose of the PCa lesions per administrated activity was 6.00E-2 ± 4.74E-2mGy/MBq. Indeed, for an administered activity of 3.7 GBq, the mean dose absorbed by the lesion would be 0.22 Gy.

**Conclusions:**

Dosimetry showed that the dose absorbed by PCa recurrences/metastases per administrated activity was low. The dosimetric study performed does not take into account the possible therapeutic effect of the Auger electrons. Clinical trials are needed to evaluate ^64^Cu internalization in the cell nucleus that seems related to the therapeutic effectiveness reported in preclinical studies.

**Electronic supplementary material:**

The online version of this article (10.1186/s13550-018-0373-9) contains supplementary material, which is available to authorized users.

## Background

Copper is an essential trace element that plays a fundamental role in a series of critical biochemical pathways. The metabolism of copper involves various transporters and copper-binding proteins; among these, the human copper transporter 1 (hCTR1) has been identified as the main mediator of copper uptake by cells [1–3]. Scientific evidence has shown that the metabolism of this element is markedly altered in neoplastic diseases [4–11]. Indeed, elevated levels of copper have been found in a wide range of tumour tissues (i.e. glioma, breast, gynaecological, gastric, bowel, lung and prostate neoplasms) [12–16]. Natural copper comprises two stable isotopes (^63^Cu and ^65^Cu) and 27 known radioisotopes, five of which are particularly promising for molecular imaging applications (^60^Cu, ^61^Cu, ^62^Cu and ^64^Cu) and two for in vivo targeted radiation therapy (^64^Cu and ^67^Cu) [7]. The versatility of ^64^Cu, which decays via three processes, namely positron, electron capture and beta decays (*T*_1/2=_12.7 h; *E*_mean β+_ = 0.28 MeV (17.86%); *E*_mean β−_ = 0.19 MeV (39.0%); EC (43.075%) [17], makes it the most widely studied radioisotope, in the areas of both PET imaging and the targeted radiotherapy of cancer. [18]. The beta particle emission implies a high local radiation dose that is theoretically suitable for targeted radionuclide therapy. Furthermore, the electron capture decay is followed by the emission of Auger electrons characterized by high linear energy transfer (LET); this is able to considerably increase the cytotoxic power of ^64^Cu, if the radioisotope is targeted near or within the cell nucleus. Because of these properties, ^64^Cu is described as the archetypal “theranostic” radioisotope, as it is potentially useful in PET/CT imaging and in radionuclide therapy [19]. A growing body of evidence suggests that ^64^Cu may provide relevant therapeutic effects. Indeed, some preclinical in vitro and animal studies have been recently conducted on this topic [20–28]. On the other hand, little can be said about the effect of ^64^CuCl_2_ in humans, and only two preliminary reports have confirmed the therapeutic effect of ^64^CuCl_2_ in patients affected by relapsing malignancies (i.e. glioblastoma, prostate and uterine cancer) [29, 30]. However, two pioneering studies, on the diagnostic role of ^64^CuCl_2_-PET/CT in human prostate cancer, reported that this imaging procedure has high sensitivity in detecting prostate cancer relapse [4, 8]. In this field, the International Atomic Energy Agency (IAEA) has implemented a coordinated research project entitled “Copper-64 Radiopharmaceuticals for Theranostic Applications”, which is focused on the application of theranostic properties of ^64^Cu and on developing and evaluating the most promising ^64^Cu-chelated targeting agents for the therapy and diagnosis of human diseases (http://cra.iaea.org/cra/stories/2015-12-22-F22067-Copper-64-Radiopharmaceuticals.html).

In a recent prospective paper, we investigated the ability of ^64^CuCl_2_-PET/CT as a diagnostic agent to detect prostate cancer (PCa) recurrence in 50 patients with biochemical relapse, after prostatectomy or external beam radiation therapy (EBRT) [4]. In the same group of patients, we also compared the ^64^CuCl_2_-PET/CT results with those of ^18^F-Choline PET/CT and multiparametric magnetic resonance imaging (mpMRI). In parallel, we evaluated the biodistribution of ^64^CuCl_2_ and the absorbed dose to organs in all patients. The biodistribution of ^64^CuCl_2_ and the absorbed dose to organs were evaluated by considering as the source organs those with the highest uptake of ^64^CuCl_2_ (namely liver, kidneys, pancreas, gallbladder wall, salivary glands and spleen). The effective dose was also calculated by using the coefficients of radiosensitivity of the organs present in publications 60 and 103 of the International Commission on Radiological Protection (ICRP) [31, 32].

In the present study, by using the same source data, we evaluated (i) the kinetic aspects, (ii) the tumour/background ratio (TBR) as a function of time and (iii) the absorbed dose of ^64^Cu dichloride in PCa lesions.

In addition, we extended the previous evaluation [4] of the dose absorbed by the organs and the effective dose by including the analysis of the red marrow as a source organ.

## Methods

The local ethics committee and the “Agenzia Italiana del Farmaco”, a public agency of the Italian Ministry of Health, approved this study. All subjects provided written informed consent. The trial was registered in the European Clinical Trial Database (EudraCT number 2014-005140-18).

### Patient population and diagnostic protocol

The population analyzed was the same as in the previous study [4]. Fifty PCa patients were prospectively evaluated and all underwent ^64^CuCl_2_-PET/CT and mpMRI within 15 days of one another. ^64^CuCl_2_-PET/CT were acquired 1, 4 and 24 h after tracer injection [4]. The analysis presented in this paper concerns PCa lesions with a diameter larger than 1 cm (0.52 mL) detected by ^64^CuCl_2_-PET/CT; thus, 59 lesions in 35 patients were considered: 29 local recurrences, 21 lymph node metastases and 9 bone metastases. The volume mean values of the three sites of disease were 19.5 ± 25.9 mL, 4.7 ± 5.2 mL and 7.1 ± 9.7 mL, respectively.

### Lesion contouring

To evaluate kinetics and tumour dosimetry, volumes of interest (VOIs) were manually drawn by an experienced nuclear medicine physician and by one experienced radiologist on the ^64^CuCl_2_-PET/CT images acquired 1 h after tracer injection. Specifically, the VOIs were drawn after co-registration (i.e. multimodal fusion imaging) between mpMRI and PET/CT images, which was performed, only in the case of positive mpMRI findings with diameter > 1 cm, by means of dedicated software developed for research purposes (Quanta Oncology, Camelot Biomedical Systems, Genoa, Italy) [33, 34]. These VOIs were then transferred to the other two PET/CT datasets (at 4 and 24 h) of each patient, after proper co-registration with mpMRI images. Furthermore, in order to evaluate the muscle radioactivity concentration as a function of time, a spherical VOI was drawn on each PET/CT image of the upper thigh of each patient.

### Lesion kinetics

The mean concentration of ^64^CuCl_2_ radioactivity in the VOIs was recorded for all PET datasets. Time-activity curves (as a percentage of injected activity/mL) for all lesions were fitted as bi-exponential functions [35]. The effective half-life was evaluated by fitting the radioactivity concentration values of the late PET images (i.e. 4 and 24 h after injection) with a mono-exponential curve. Standard uptake values (SUVs, i.e. SUV_mean_ and SUV_max_) in all VOIs were also recorded, and their values were recalculated from their respective kinetic curves at the reference time of 1 h after administration. Furthermore, the mean concentration values for tumours, the main organs at risk (namely liver, kidneys and pancreas) and the muscle were calculated from the corresponding kinetic curves at 1, 4 and 24 h after administration. To conduct the analysis of the organs at risk, the kinetic curves obtained in the previous study were used [4].

In order to take into account the partial volume effect (PVE), in our study, the recovery correction method was used [36–39]. This method is based on numerical coefficients (recovery coefficients, RCs) that are experimentally measured by using radioactive phantoms. RCs are used to recover the radioactivity concentration measured by the PET tomograph and are obtained as the ratio between the PET-measured radioactivity concentration and the actual radioactivity concentration within the hot spheres simulating PCa lesions. To this end, the six spheres of various diameters (10, 13, 17, 22, 28 and 37 mm), which are part of the NEMA IEC Body Phantom insert (http://www.spect.com/pub/NEMA_IEC_Body_Phantom_Set.pdf), were filled with a known concentration of ^64^CuCl_2_. To simulate the background, the body phantom chamber was also filled with radioactivity. Three different phantom TBR (3, 9 and 20) were chosen in order to reproduce the clinical range patients’ tumour/background ratio (namely about 2–15). The PET/CT acquisition of the phantom was performed by using the same clinical protocol setup described in the previous study [4]. Six spherical VOIs, having the same diameters of the spheres of NEMA phantom, were drawn on the corresponding spheres on PET/CT images. In order to calculate RC values, the mean radioactivity concentration was measured in each VOI by means of PMOD software (PMOD, Zurich, Switzerland). The RC curves (Additional file 1: Figure S1) were obtained by fitting RC experimental data with the function:

1 $$ \mathrm{RC}=\alpha \cdotp {\left(\frac{V}{V+\beta}\right)}^{\gamma } $$

where *V* is the volume of the sphere (*α*, *β* and *γ* are the fit parameters).

### Statistical analysis

Continuous and categorical factors were described by using mean, standard deviation (SD), quartiles, minimum, maximum and absolute/relative (%) frequencies, respectively. Box plots were adopted to visually describe data, and differences among groups were tested by using non-parametric tools (Kruskal-Wallis equality-of-populations rank test and Mann-Whitney test). No multiple testing adjustments were applied. All *p* values (two-sided) < 0.05 were considered statistically significant. The statistical software adopted was STATA v.14.2 (College Station, TX, USA).

### Tumour/background ratio

The TBR of all lesions was evaluated. The background radioactivity concentration was obtained by calculating the mean value of four VOIs drawn at 1 cm from each lesion. Furthermore, to evaluate the TBR_max_ and TBR_mean_ of ^64^CuCl_2_-PET/CT, the three values of TBR were fitted with a bi-exponential curve as a function of time. The mean TBR curve was obtained by averaging the TBR fitting curves of all tumours in each site of disease.

### Tumours and red marrow dosimetry

To perform the dosimetry of the PCa lesions, the accumulated activity (the sum of all nuclear transitions that occur inside the tumour) for all lesions was calculated as the area under the time-activity curve, and the time-integrated activity coefficient (*τ*) was obtained dividing the accumulated activity by the administered activity. The activity considered in the tumours was corrected for the PVE. The absorbed dose per administrated activity for each lesion was calculated by using the Medical Internal Radiation Dose (MIRD) system [40, 41]. Using OLINDA/EXM software [42], S-factors for spheres for ^64^Cu (in mGy/MBq*h) were obtained by fitting tumour masses (*m*, in grams), in the range of our study, with the function (Additional file 2: Figure S2):

2 $$ S=\frac{A}{m^B} $$

where *A* and *B* are the fit parameters.

For local and lymph node lesions, a density of 1 g/mL was considered, while for bone tumours, a mean density of 1.2 g/mL was estimated from CT Hounsfield numbers.

To calculate the dose absorbed by the red marrow, VOIs corresponding to L4 and L5 were drawn in each ^64^CuCl_2_-PET/CT dataset for every patient (50 in total) [43]. Indeed, the red marrow in these two vertebrae accounts for 6.8% of that of the entire skeleton [44]. Thus, the number of disintegrations in this vertebral region was divided by 0.068 to obtain the number of disintegrations in the entire red marrow.

## Results

### Lesion kinetics

**Fig. 1:**
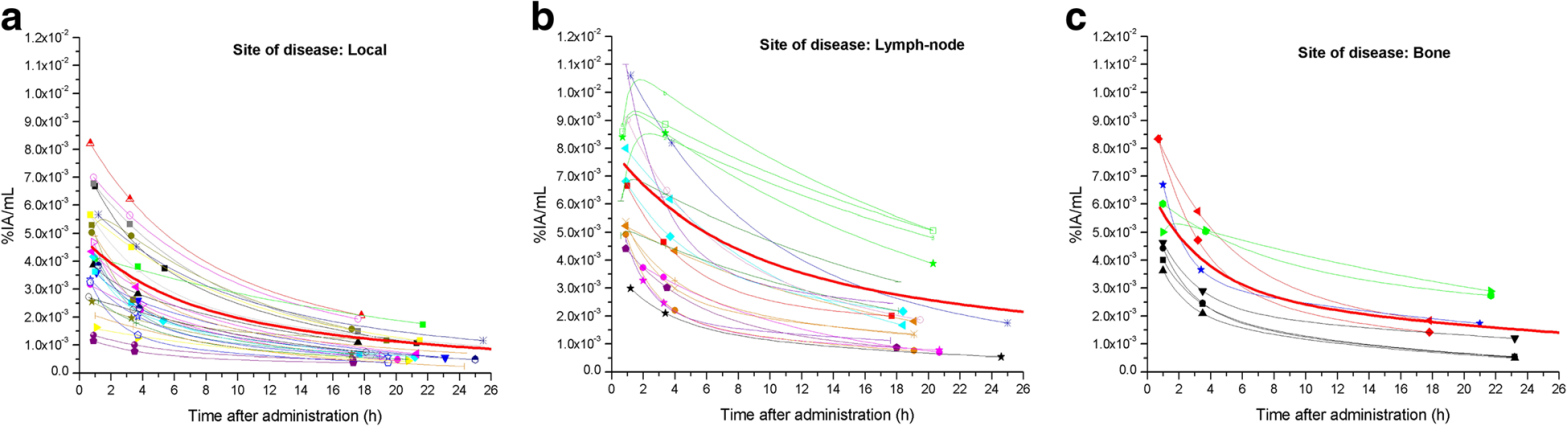
PVE corrected time-activity curves of ^64^CuCl_2_ (as a percentage of injected activity/mL) for the three different site of disease: local (**a**), lymph node (**b**) and bone (**c**) (in red the mean curves)

**Fig. 2:**
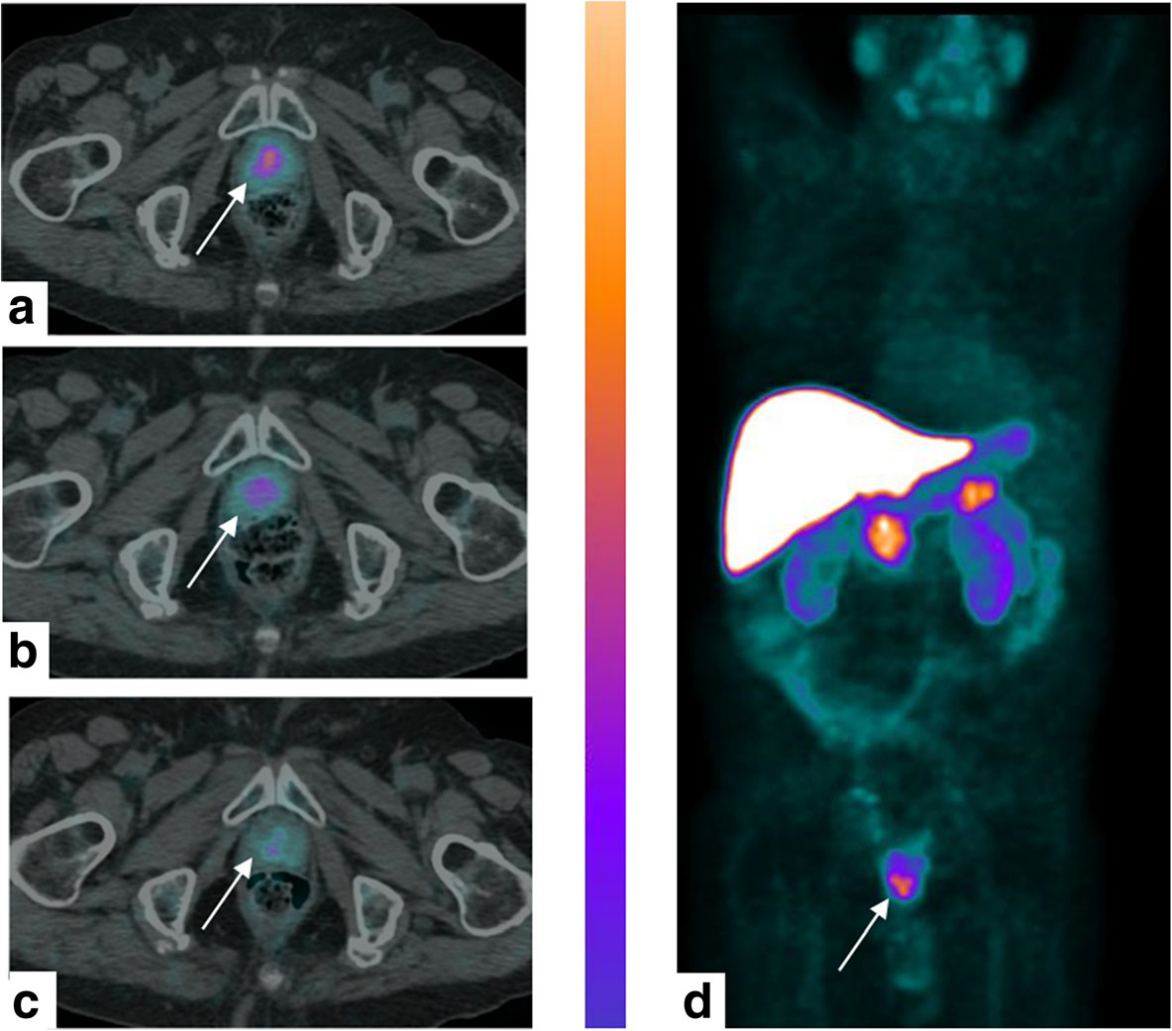
An 85-year-old man affected by Gleason 4+3 PCa treated with EBRT, with rising PSA level (6.0 ng/mL) and PSA doubling time of 15 months. ^64^CuCl_2_-PET/CT images (axial and MIP) revealed focal and pathologic tracer uptake (arrows) in correspondence to the prostate apex close to the midline 1 h after the injection (**a**). An important reduction of tracer uptake was observed 4 (**b**) and 24 h (**c**) after the injection. Maximum intensity projection (**d**) visualized the differences in terms of uptake between the organsand prostate relapse

**Fig. 3:**
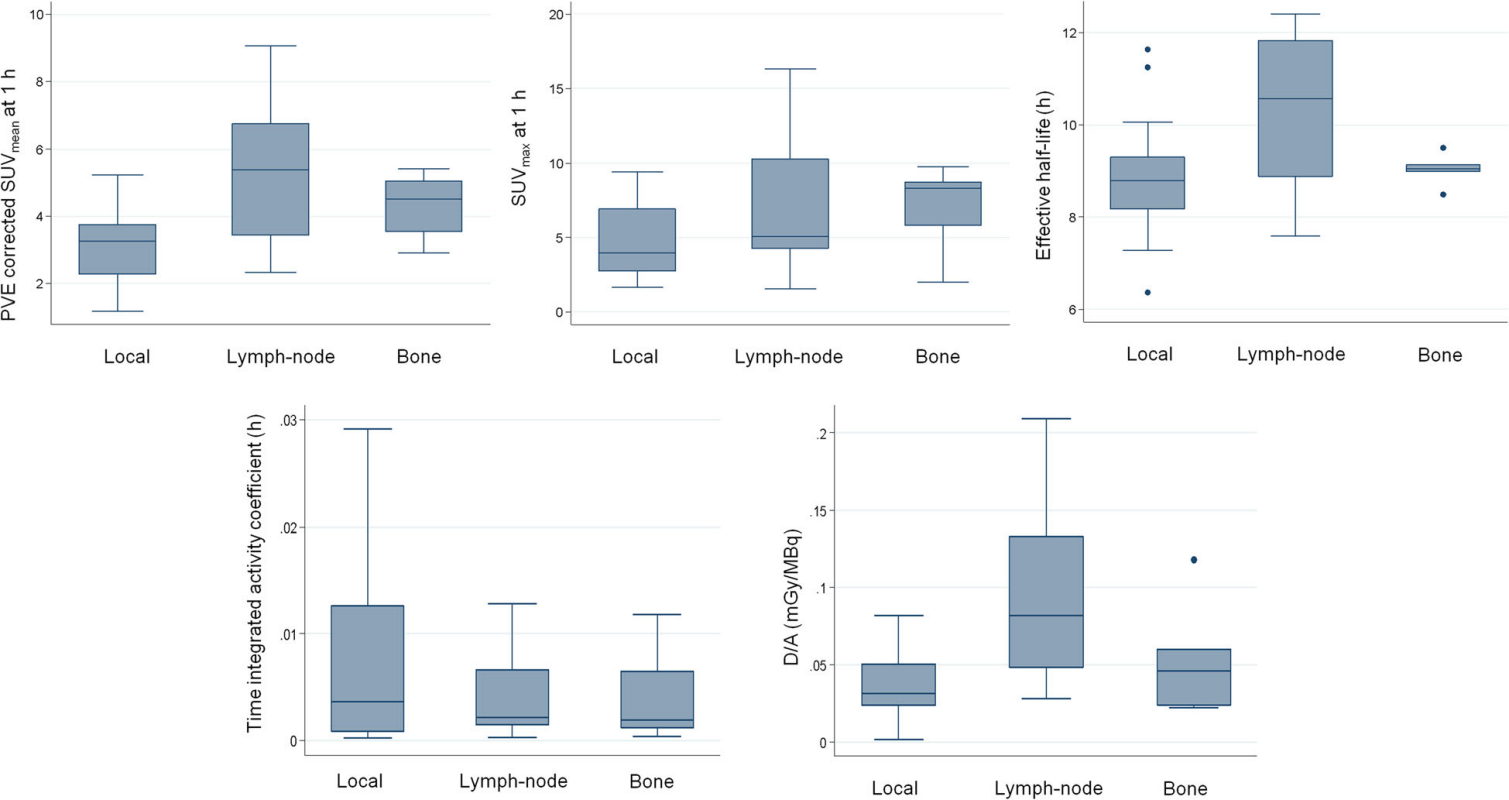
Box plot representation of PVE corrected SUV_mean_ and SUV_max_ values 1 h after tracer injection, the effective half-life, the time-integrated activity coefficient and the absorbed dose (per administrated activity) for the three sites of disease

**Fig. 4:**
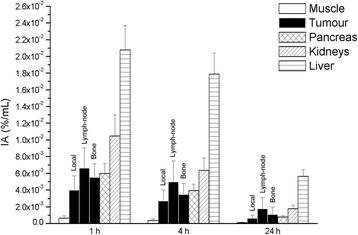
Specific concentration (as a percentage of injected activity) for lesions (for each site of disease) and the main organs at risk 1, 4 and 24 h after administration

PVE-corrected time-activity curves of ^64^CuCl_2_ for the three types of lesion (local relapses and lymph node and bone metastases) showed that the uptake was rapid, reaching the maximum value approximately 1 h after ^64^CuCl_2_ administration (Fig. 1). The bi-exponential fitting curves highlighted a rapid clearance in the first few hours after the maximum uptake, followed by a slower tracer elimination starting from the fourth hour. The trend of ^64^CuCl_2_ uptake over time is illustrated in Fig. 2. Table 1 summarizes the differences in mean effective (and biological) half-life, the PVE-corrected SUV_mean_ and SUV_max_ mean values 1 h after ^64^CuCl_2_ injection, the time-integrated activity coefficients and the absorbed dose (per administrated activity), among the three sites of PCa relapse. A box plot representation of these parameters is shown in Fig. 3. A statistically significant difference in terms of effective half-life was observed between local recurrences and lymph node metastases. We also found a significant difference in terms of PVE-corrected SUV_mean_ and SUV_max_ evaluated 1 h after tracer injection between local and lymph node metastases and between local recurrences and bone metastases. Furthermore, a statistically significant difference in terms of absorbed dose was observed between local relapses and lymph node metastases (Table 1). In Fig. 4, the ^64^CuCl_2_ concentration (as a percentage of injected activity) in the PCa lesions, for each site of diseases, and in several organs is shown. Over time, the tumour concentration was about 4.5 and 2.0 times lower than in the liver and kidneys, respectively.

**Table 1:** Summary table of the main tumour results

	Local recurrences	Lymph node metastasis	Bone metastasis	*p* Local vs lymph node	*p* Local vs bone	*p* Lymph node vs Bone
T_1/2eff_ (T_1/2biol_) (h)	8.8 ± 1.1 (28.7 ± 3.6)	10.2 ± 1.7 (51.8 ± 8.6)	9.0 ± 0.4 (30.9 ± 1.4)	**0.003**	0.9	0.4
SUV_mean_ at 1 h	3.1 ± 1.1	5.2 ± 2.1	4.3 ± 0.9	**< 0.001**	**0.003**	0.8
SUV_max_ at 1 h	4.7 ± 2.4	6.8 ± 4.3	6.8 ± 2.9	**0.02**	**0.03**	0.5
τ (h)	1.1E-2 ± 2.4E-2	6.1E-3 ± 7.8E-3	6.4E-3 ± 1.0E-2	0.3	0.2	0.8
D/A (mGy/MBq)	3.71E-2 ± 1.87E-2	9.65E-2 ± 5.95E-2	5.40E-2 ± 3.86E-2	**< 0.001**	0.1	0.6

Values are reported as mean ± standard deviation. Statistically significant differences (*p* < 0.05) are in bold

### Tumour/background ratio

**Fig. 5:**
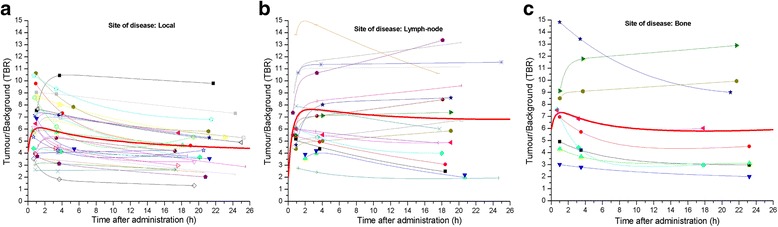
^64^CuCl_2_ TBR curves over the time for the three different site of disease: local (**a**), lymph node (**b**) and bone (**c**) (in red the mean curves)

Background time-activity curves of ^64^CuCl_2_ showed the same bi-exponential trend as the tumour time-activity curves. The maximum uptake was observed about 1 h after ^64^CuCl_2_ administration. The typical time-activity curves of PCa relapse and background are illustrated in Additional file 3: Figure S3. The ^64^CuCl_2_-PET TBR curves of the local recurrences and lymph node and bone metastases are illustrated in Fig. 5. These curves revealed that the mean TBR value (TBR_mean_) was 5.0, 7.0 and 6.2 over 24 h, and the TBR_max_ was 6.1, 7.6 and 7.4 for local relapses and lymph node and bone metastases, respectively. The TBR_max_ was achieved about 1 h after radiopharmaceutical administration for each site of disease.

### Tumours and red marrow dosimetry

**Fig. 6:**
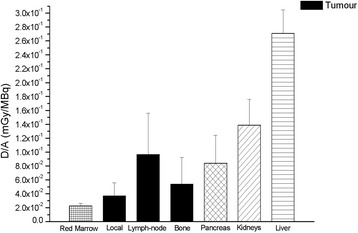
Absorbed dose per administrated activity in PCa lesions (for each site of disease), red marrow, pancreas, kidneys and liver

The mean dose absorbed by PCa lesions per administered activity was 6.00E-2 ± 4.74E-2mGy/MBq (3.71E-2 ± 1.87E-2 mGy/MBq, 9.65E-2 ± 5.95E-2 mGy/MBq and 5.40E-2± 3.86E-2mGy/MBq for local relapses and lymph node and bone metastases, respectively. The maximum uptake by red marrow was about 2% of the injected activity, and the mean absorbed dose per administered activity was 2.26E**-**2 ± 9.04E-3 mGy/MBq. Thus, the effective dose was 3.10E-2 ± 8.07E-3 mSv/MBq and 2.91E-2 ± 7.83E-3 mSv/MBq when it was recalculated by using the radio-sensitivity coefficients of the organs of the ICRP protocols 60 and 103, respectively [31, 32]. The recalculated doses absorbed by the organs are reported in Table 2. In Fig. 6, the mean absorbed dose by each site of diseases and by several organs is shown. Radiation dosimetry analysis showed that the mean absorbed dose per administered activity was 4.5 and 2.3 times higher in the liver and kidneys, respectively, than in PCa lesions.

**Table 2:** Absorbed organs dose per administered activity (± SD) for ^64^CuCl_2_

Organ	Absorbed organ dose per administered activity (mGy/MBq)
Adrenals	2.54E-2 (± 6.60E-3)
Brain	1.04E-2 (± 3.95E-3)
Breasts	1.22E-2 (± 4.07E-3)
Gallbladder wall	7.82E-2 (± 2.99E-2)
Lower larger intestine wall	1.25E-2 (± 4.76E-3)
Upper large intestine wall	1.78E-2 (± 5.31E-3)
Small intestine	1.62E-2 (± 5.42E-3)
Stomach wall	1.70E-2 (± 5.48E-3)
Heart wall	1.80E-2 (± 5.30E-3)
Kidneys	1.39E-1 (± 3.72E-2)
Liver	2.71E-1 (± 3.37E-2)
Lungs	1.64E-2 (± 4.88E-3)
Muscle	1.34E-2 (± 4.51E-3)
Pancreas	8.39E-2 (± 4.03E-2)
Red marrow	2.26E-2 (± 9.04E-3)
Osteogenic cells	3.00E-2 (± 1.16E-2)
Skin	1.09E-2 (± 3.84E-3)
Spleen	3.63E-2 (± 1.37E-2)
Testes	1.10E-2 (± 4.14E-3)
Thymus	1.31E-2 (± 4.53E-3)
Thyroid	1.16E-2 (± 4.31E-3)
Urinary bladder wall	1.27E-2 (± 4.69E-3)
Salivary glands	3.70E-2 (± 3.70E-2)
Total body	2.09E-2 (± 5.61E-3)
Effective dose ICRP 60 (mSv/MBq)	3.10E-2 (± 8.07E-3)
Effective dose ICRP 103 (mSv/MBq)	2.91E-2 (± 7.83E-3)

## Discussion

In our study, a thorough evaluation of ^64^Cu dichloride behaviour in human PCa was performed. Specifically, we analysed the kinetics and dosimetry of the three typical sites of PCa relapse: local recurrences, lymph node metastases and bone metastases. The kinetic analysis of the PCa lesions showed a rapid uptake within the first hour after administration, followed by a washout phase characterized by two different biological times: rapid clearance in the first hour after the maximum uptake and a slower kinetic starting from the fourth hour after the tracer injection. This behaviour was also found in the muscles and in the tissues around the tumours (i.e. background) and had already been observed in the organs in our previous study [4]. On the one hand, the time to maximum uptake was similar in both healthy and pathologic tissues. On the other hand, the effective half-life was different in each healthy tissue and in each different site of disease relapse. Indeed, the ^64^CuCl_2_ half-life in lymph node metastases was significantly longer than in local recurrences. When the uptake intensity of ^64^CuCl_2_ was considered, lymph node metastases and bone metastases showed significantly higher SUV_mean_ and SUV_max_ mean values than local recurrences. Our study highlighted that the mean concentration of ^64^CuCl_2_ in PCa lesions over time was about 4.5 and 2.0 times lower than in the liver and kidneys, respectively. This result is consistent with the values already published in mice with regard to melanoma, prostate cancer and hepatocellular carcinoma [9–11]. Therefore, it can be hypothesized that this behaviour is independent of the tumour subtype.

The high ^64^CuCl_2_ TBR in PCa recurrences/metastases, as confirmed in this study, is the most important characteristic of this tracer. Indeed, the high contrast between the lesions and the surrounding healthy tissue may considerably improve the diagnostic performance of PET/CT. On evaluating the TBR curves, we found that the ^64^CuCl_2_ TBR_max_ was reached approximately 1 h after tracer injection.

To our knowledge, this is the first study to investigate the ^64^CuCl_2_ dosimetry of human PCa. The mean dose absorbed by the tumors per administered activity, calculated by means of the MIRD, was 6.00E-2 mGy/MBq. This absorbed dose was 4.5 and 2.3 times lower than the mean values in the liver and kidneys, respectively. For an administered activity of 3.7 GBq, the mean dose absorbed by the lesions would be 0.22 Gy. This low value seems to suggest the hypothesis that the reported therapeutic effect of ^64^CuCl_2_ [20–30] is mainly dependent on Auger electron emission rather than on the energy released by the beta radiation. In this setting, to be effective, ^64^Cu should be internalized in the cell nucleus [45–47], since Auger cascade electrons have an energy spectrum dominated by a large number of very low energy electrons with a very narrow range in biological matter. Indeed, cellular and organ studies have demonstrated that when Auger emitters are introduced into the cytoplasm of cells, the effects are typical of those caused by radiation of low LET, such as photons and beta radiations [48]. On the other hand, when Auger emitters are incorporated into the cell nucleus, the LET of these electrons can cause similar biological damage to that elicited by heavier particles with high LET, such as alpha particles [49]. Therefore, the cytocidal effect of the Auger electrons of ^64^Cu appears to be much more severe than that of beta emission; indeed, some studies report effects up to 5–25 times [50–61]. In addition, since the liver has been identified as the organ receiving the higher dose, owing to its high uptake [4, 62, 63], it is important to understand whether ^64^Cu is internalized in the nucleus of hepatic cells or not. If it is not, the potential hepatic radiotoxicity might be induced by the beta emission component only by means of very high injected activity [4]. More detailed radiobiological studies are required to characterize the radiotoxicity and therapeutic effects of ^64^CuCl_2_. However, to really understand whether or not the liver is the critical organ in the case of therapeutic application of ^64^CuCl_2_, phase I clinical trials, not available at the moment, should be conducted.

Since the red marrow is a dose-limiting organ in radionuclide therapy, we extended the dosimetry performed in the previous study [4] and included the red marrow as a source organ, too. We found that the absorbed dose in the red marrow was 2.26E-2 mGy/MBq, i.e. 75% higher than that of previously published [4]. As expected, the absorbed doses in other organs were substantially unvaried.

Despite our encouraging results, at least few limitations should be taken into account. The lack of dynamic acquisition in the uptake phase implies poor knowledge of the radionuclide behaviour immediately after administration. Furthermore, the acquisition of other time points would let better time-integrated activity coefficient and dosimetry evaluations. Finally, the absorbed dose by the red marrow was not calculated using blood sampling. However, to facilitate patient compliance, we decided not to insist on these long diagnostic procedures.

## Conclusions

The high diagnostic sensitivity of ^64^CuCl_2_ PET/CT is related to its high TBR_max_, such a value was reached about 1 h after administration. Dosimetry based on MIRD showed that the dose absorbed by PCa recurrences/metastases per administrated activity was low and unlikely able to reach therapeutic effects. Clinical trials are needed in order to evaluate ^64^Cu internalization in the cell nucleus that seems related to the therapeutic effectiveness reported in preclinical studies.

## Additional files

Additional file 1: **Figure S1.** Experimental RC values for three TBR values. (TIFF 405 kb)

Additional file 2: **Figure S2.** S-factors specific for variable mass spheres for ^64^Cu (OLINDA/EXM software). (TIFF 381 kb)

Additional file 3: **Figure S3.** Typical time-activity curve for tumour and background. (TIFF 397 kb)

## Abbreviations

EBRT: External-beam radiation therapy
hCTR1: Human copper transporter 1
IAEA: International Atomic Energy Agency
ICRP: International Commission on Radiological Protection
LET: Linear energy transfer
MIRD: Medical Internal Radiation Dose
mpMRI: Multiparametric magnetic resonance imaging
PCa: Prostate cancer
PVE: Partial volume effect
RC: Recovery coefficient
SD: Standard deviation
SUV: Standard uptake value
TBR: Tumor/background ratio
VOI: Volume of interest
