# Supplementary figures and images for: Biokinetic and dosimetric aspects of 64CuCl2 in human prostate cancer: possible theranostic implications
Source: EJNMMI Res. 2018 Mar 1;8:18. doi: 10.1186/s13550-018-0373-9 (PMC5833894; doi:10.1186/s13550-018-0373-9)

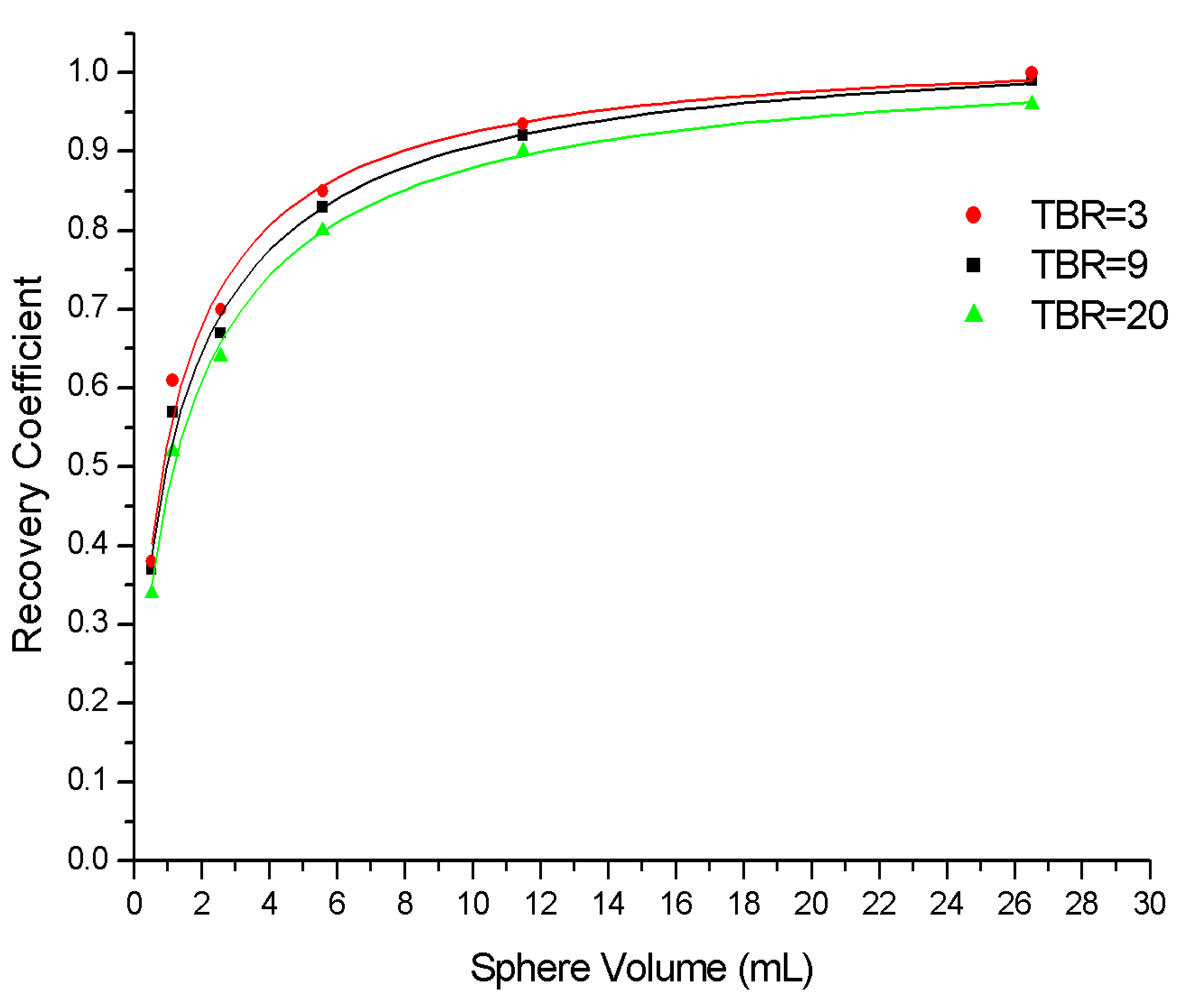

Supplement: Supplementary file 1 — Figure S1. Experimental RC values for three TBR values. (TIFF 405 kb) [file 13550_2018_373_MOESM1_ESM.tif]

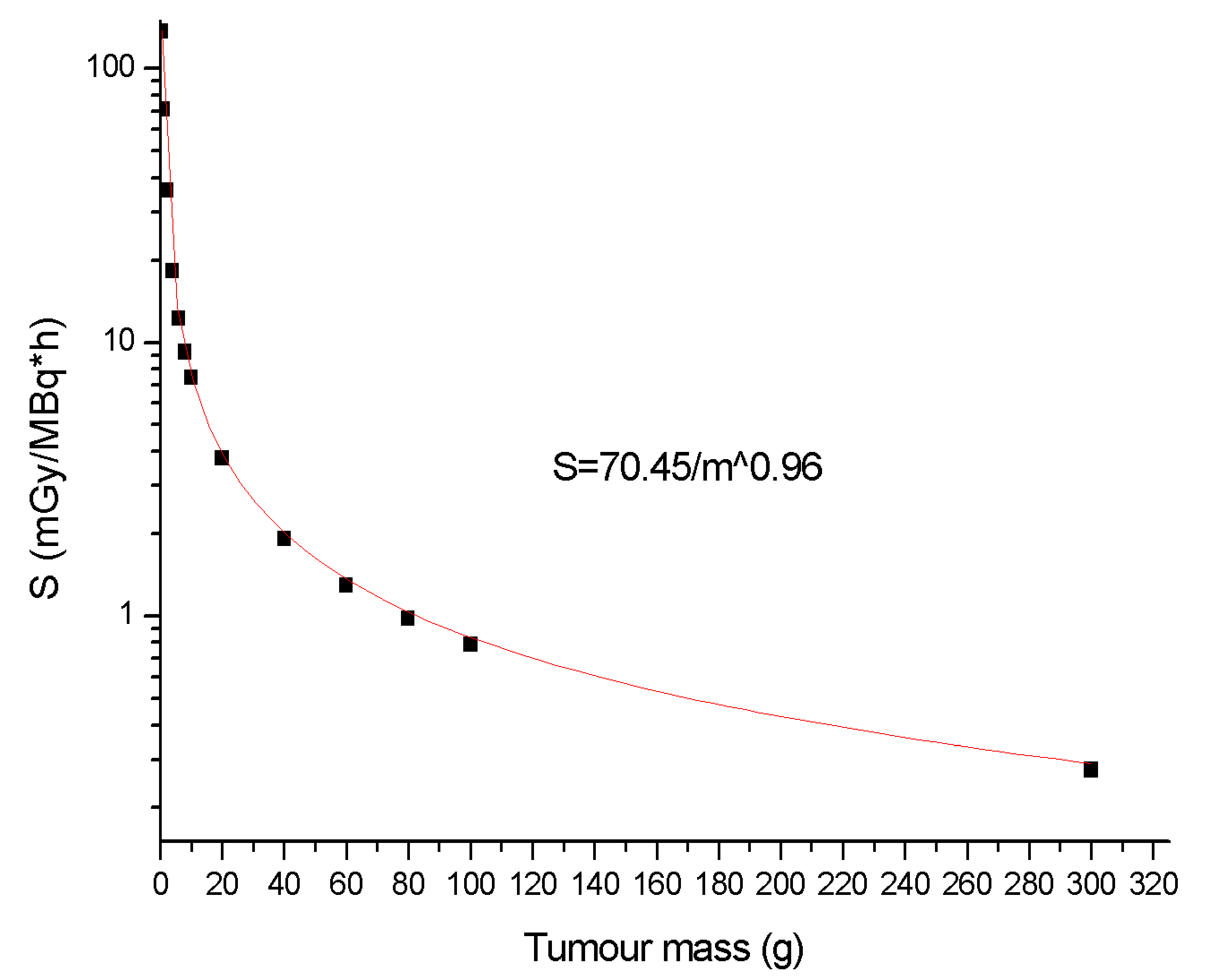

Supplement: Supplementary file 2 — Figure S2. S-factors specific for variable mass spheres for 64Cu (OLINDA/EXM software). (TIFF 381 kb) [file 13550_2018_373_MOESM2_ESM.tif]

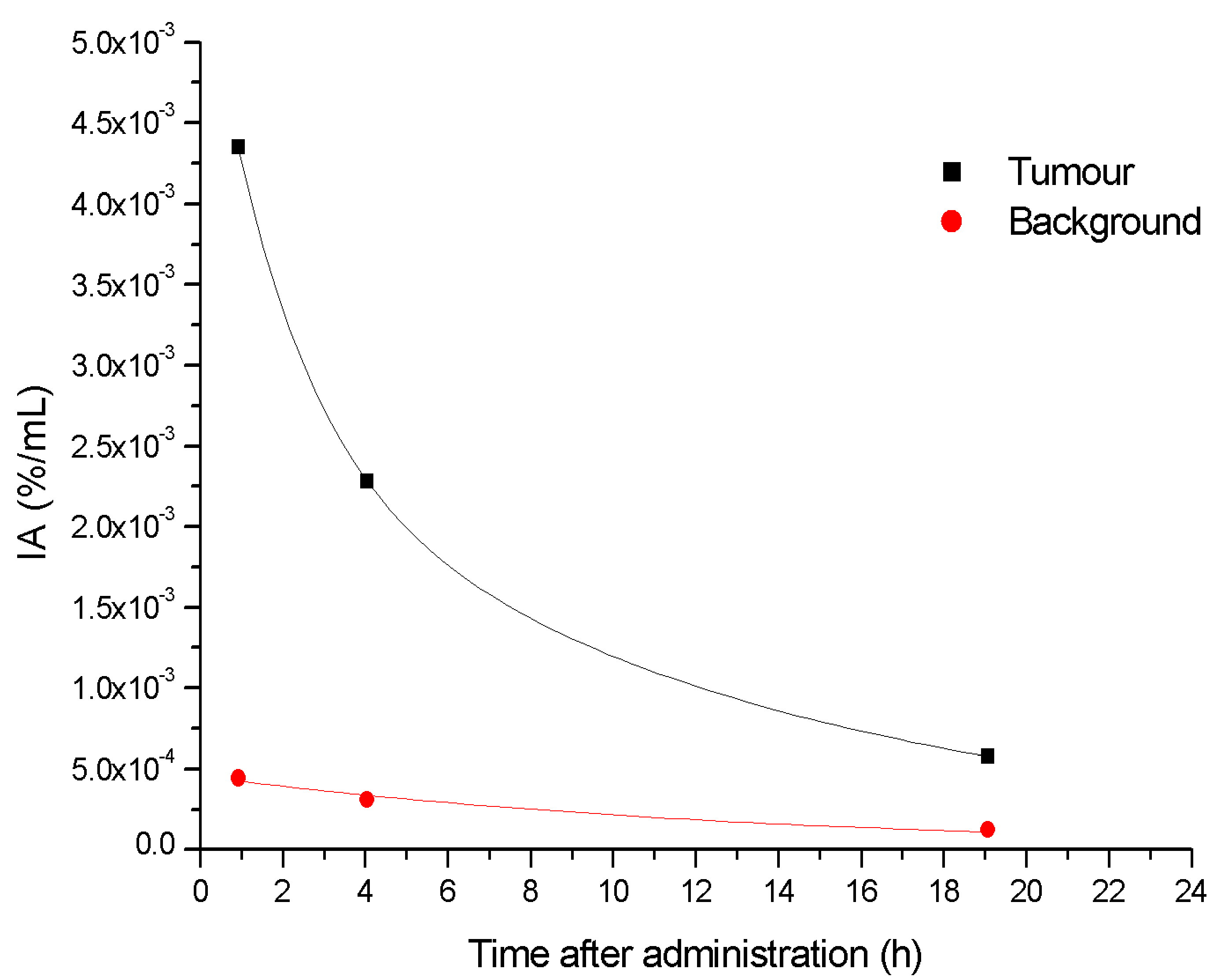

Supplement: Supplementary file 3 — Figure S3. Typical time-activity curve for tumour and background. (TIFF 397 kb) [file 13550_2018_373_MOESM3_ESM.tif]
